# Chronic viral infection compromises the quality of circulating mucosal-invariant T cells and follicular T helper cells via expression of both activating and inhibitory receptors

**DOI:** 10.21203/rs.3.rs-2862719/v1

**Published:** 2023-04-27

**Authors:** Jaisheela Vimali, Yean Kong Yong, Amudhan Murugesan, Hong Yien Tan, Ying Zhang, Rajeev Ashwin, Sivadoss Raju, Pachamuthu Balakrishnan, Marie Larsson, Vijayakumar Velu, Esaki M Shankar

**Affiliations:** Department of Life Sciences, Central University of Tamil Nadu, Thiruvarur, India; Xiamen University, Sepang, Malaysia; Department of Microbiology, Government Theni Medical College and Hospital, Theni, India; Xiamen University, Sepang, Malaysia; Xiamen University, Sepang, Malaysia; Department of Life Sciences, Central University of Tamil Nadu, Thiruvarur, India; Directorate of Public Health and Preventive Medicine, Chennai, India; Centre for Infectious Diseases, Saveetha Dental College and Hospitals, Saveetha Institute of Medical and Technical Sciences (SIMATS), Chennai, India; Division of Molecular Medicine and Virology, Department of Biomedical and Clinical Sciences, Linköping University, 58185 Linköping, Sweden; Department of Pathology and Laboratory Medicine, Emory National Primate Research Center, Emory University, Atlanta GA, United States; Department of Life Sciences, Central University of Tamil Nadu, Thiruvarur, India

**Keywords:** HBV, HIV, MAIT cells, PD-1, T cell exhaustion

## Abstract

Chronic viral infection results in impaired immune responses rendering viral persistence. Here, we investigated the role of immune activation and compared the quality of T-cell responses in chronic HBV, HCV, and HIV infections. Cytokines were measured using a commercial Bio-plex Pro Human Cytokine Grp I Panel 17-plex kit (BioRad, Hercules, CA, USA). Inflammation was assessed by measuring an array of plasma cytokines, and peripheral CD4^+^ T cells including circulating Tfh cells, CD8^+^ T cells, and TCR iVα7.2^+^ MAIT cells in chronic HBV, HCV, and HIV-infected patients and healthy controls. The cells were characterized based markers pertaining to immune activation (CD69, ICOS, and CD27) proliferation (Ki67), cytokine production (TNF-α, IFN-γ) and exhaustion (PD-1). The cytokine levels and T cell phenotypes together with cell markers were correlated with surrogate markers of disease progression. The activation marker CD69 was significantly increased in CD4^+ hi^ T cells, while CD8^+^ MAIT cells expressing IFN-γ were significantly increased in chronic HBV, HCV and HIV infections. Six cell phenotypes, viz., TNF-α^+^CD4^+ lo^ T cells, CD69^+^CD8^+^ T cells, CD69^+^CD4^+^ MAIT cells, PD-1^+^CD4^+ hi^ T cells, PD-1^+^CD8^+^ T cells, Ki67^+^CD4^+^ MAIT cells were independently associated with decelerating the plasma viral load (PVL). TNF-α levels showed a positive correlation with increase in cytokine levels and decrease in PVL. Chronic viral infection negatively impacts the quality of peripheral MAIT cells and TFH cells via expression of both activating and inhibitory receptors.

## Introduction

Chronic viral infections results in immune cell dysfunctions in the host [1], but often persist without inflicting any serious cell damage [2]. Many chronic viruses, especially the hepatitis B virus (HBV), hepatitis C virus (HCV) and human immunodeficiency virus 1 (HIV-1) in humans are adept at circumventing the host’s immune responses, primarily by imposing the expression of co-inhibitory molecules to the advantage of the pathogen [3]. Given the non-cytopathic nature of HBV and HCV, the immune system is attributed to hepatocellular damage as well as viral clearance [4]. Inability to attain viral clearance and development of chronic HBV disease is suggestive of dysfunctional immune responses [5]. The expansion of regulatory T cells (Tregs), high antigen loads, anti-inflammatory cytokines, and biosignatures of exhaustion are the likely indications of dysfunctional HBV-specific responses [6]. Evasion of the host’s immune surveillance augments active replication of chronic viruses. Besides, viral persistence also stems from clonal deletion of HBV-specific T cells and/or their functional insufficiency together with increased expression of signatures associated with immune activation, senescence, and exhaustion.

Chronic HCV infection leads to persistent upregulation of genes associated with innate immune activation leading to liver inflammation and consequently cirrhosis [7]. Exhausted T cells in concert with TNF-α and IFN-γ, are capable of driving non-specific immune responses in order to prolong the infection [8]. During HIV infection, it’s clear that the virus dominates with the loss of immunological control over viral replication in treatment-naïve individuals. Exhausted virus-specific CD8^+^ T cells progressively lose their ability to clear cellular reservoirs of viruses [9], due to chronic immune activation that results in functional immune exhaustion [10]. MAIT cells appear to undergo functional impairment during chronic infections. The current research intends to generically and descriptively determine the quality of host immune responses in chronic HBV, HIV, and HCV infections. We investigated the role of immune activation and potentially compromised T-cell responses in the three different chronic viral infections by exploring conventional CD4^+^ and CD8^+^ T cells along with their counterpart follicular T helper cells (Tfh) and mucosal-associated invariant T cells (MAIT).

## Materials and methods

### Ethics approval

The study was carried out in accordance with the guidelines of the International Conference on Harmonization Guidelines and the Declaration of Helsinki. The study protocols were reviewed by the Institutional Ethical Committee (IEC) of the Government Medical College, Theni, for necessary approval for the conduct of the research (Ref. No. 2544/ME1/18 and Ref. No. 1515/MEIII/21). Institutional Biosafety Committee (IBSC) approval was secured (Ref. No.: CUTN/SLS/1st IBSC/2020/04). All the human subjects were adults and written consents were duly obtained from all the participants.

### Subjects and analytical parameters

HBV-infected individuals with plasma HBsAg and anti-HBc positivity (n = 13), HCV-infected individuals as determined by anti-HCV (n = 8), HIV-infected individuals (as per the criteria of the National AIDS Control Organization (NACO), India) (n = 7), and healthy controls (HCs) (n = 10) were recruited into the cross-sectional study. Peripheral blood was obtained from all the participants by a trained phlebotomist. HCs were identified as individuals free from HBV, HCV, HIV and *Mycobacterium tuberculosis infections*.

Plasma aspartate aminotransferase (AST), alanine aminotransferase (ALT), γ-glutamyl transferase (GGT), and alkaline phosphatase (ALP) levels were measured on a Semi-automatic Analyzer (Rapid Diagnostics Star 20, Hyderabad, India) using commercial kits procured from TRUEchemie, Brussels, Belgium; AST assay kit (Lot. No. A1921102) with the cut-off values set at 33U/L, ALT assay kit (Lot. No. A1521101) with the cut-off values set at 35U/L, GGT assay kit (Lot. No. G1221122), with the cut-off values set at 49U/L (male) and 32U/L (female), ALP assay kit (Lot. No. A1321111) with the cut-off values set at 141U/L, respectively.

### HIV diagnosis and absolute CD4^+^ T-cell counts

HIV infection was diagnosed using the conventional three-kit method advocated by the NACO. According to the manufacturer’s instructions, the rapid immunochromatographic tests Comb Aids-RS (Arkray Healthcare, Mumbai, India), VoXpress HIV-1/2 (Voxtur Bio, Mumbai, India), and Meriscreen HIV 1–2 WB (Merillife, India) were employed for diagnosing HIV infection. At inclusion, all HIV-positive individuals were receiving active antiretroviral therapy (ART). According to the manufacturer’s instructions, anti-CD45-PE-Cy5 (Cat. No. 05–8405-02) and anti-CD4-PE (Cat. No. 05–8405-01) (Sysmex Partec GmbH, Gorlitz, Germany) fluorochrome-tagged antibodies were used for the immunophenotyping. For absolute CD4^+^ T-cell counts, 2ml of whole blood in EDTA tubes was used.

### Plasma viral load

The Pathodetect^™^ (Mylab Discovery, Pune, India) quantitative Real-Time PCR was used to quantify the viral loads of HBV and HCV using an *in vitro* nucleic acid amplification assay on a QuantStudio 5 real-time PCR (Applied Biosystems, ThermoFisher Scientific, MA, USA).

### Luminex Bio-Plex cytokine array

Cytokines were measured using the Bio-plex Pro Human Cytokine Grp I Panel 17-plex kit (BioRad, Hercules, CA, USA) that quantifies the levels of MCP-1, G-CSF, GM-CSF, IL-7, IL-12(p70), IL-1β, MIP-1β, TNF- α, CXCL8 (IL-8), IFN-γ, IL-6, IL-2, IL-4, IL-5, IL-13, IL-17 and IL-10 following the manufacturer’s instructions.

### Peripheral blood mononuclear cells

Ten milliliters of peripheral blood were collected by venipuncture, and stored in lithium heparin BD Vacutainer (BD Biosciences, Franklin Lakes, NJ, USA) tubes at room temperature. PBMCs were extracted using a commercial Sepmate^™^ (Stemcell Technologies, Vancouver, Canada) by density gradient centrifugation. Cell viability was determined by 0.4% Trypan blue vital staining. Purified PBMCs were suspended in a Bambanker^™^ serum-free cell freezing medium (Nippon Genetics Europe GmbH, Duren, Germany) for storage at −80°C. PBMCs were thawed in a water bath at 37°C before use in the experiments.

### Flow cytometry

#### Multi-parametric immunophenotyping

All antibodies were purchased from BD Pharmingen^™^ (BD Biosciences) unless otherwise specified. Immunostaining was performed with one panel each for MAIT cells, Tfh cells, and along with various markers. The MAIT cell panel included allophycocyanin H7 (APC-H7)–conjugated anti–CD3, brilliant violet 510 (BV510) anti–CD4, fluorescein isothiocyanate (FITC)–conjugated anti–CD8, phycoerythrin (PE)–conjugated TCR iVα7.2, brilliant violet 421 (BV421) conjugated anti–CD56, peridinin chlorophyll protein (PerCP)-Cy5.5–conjugated anti–ICOS, Alexa 647 anti–PD-1, PE-Cy7 anti–CD69. The TFH cell panel was performed with APC H-7 conjugated anti–CD3, BV421-conjugated anti–CXCR5, Alexa647-conjugated anti–PD-1, BB515 conjugated anti–ICOS, PE–Cy7-conjugated anti–CD27.

#### Intracellular cytokine staining

Mononuclear cells were incubated with PMA (50 ng/mL) and ionomycin (500ng/mL) or cultured in RPMI containing 10% FBS (R10) alone. Samples were incubated at a concentration of 10 µg/mL, and Golgi Plug (brefeldin A) and Golgi Stop (monensin) were included at 10 µg/mL. Samples were incubated for overnight at 37°C in 5% CO2 and then permeabilized using Fix & Perm reagents (BD Bioscience) and stained intracellularly with anti–IFN-γ (clone B27) and anti–TNF-α (clone Mab11). At the end of stimulation, cells were washed once with FACS wash (PBS containing 2% [vol/vol] FBS and 0.25% of sodium azide) and surface stained with anti-CD3, anti-TCR7.2 (3C10), anti-Ki67 (B56) anti-CD8 (SK1), anti-CD4 (OKT4) Cell stain at room temperature for 30 minutes. Cells were then fixed with Cytofix/Cytoperm (BD Pharmingen) for 20 minutes at 4°C and washed with Perm wash (BD Pharmingen). Cells were then incubated for 30 minutes at 4°C with antibodies specific to IFN-γ and TNF-α, washed once with Perm wash and once with FACS wash, and resuspended in PBS containing 1% formalin. Cells were acquired on a BD FACS Canto II Immunocytometric system. FlowJo for Windows, Ver.10.0.8 (FlowJo LLC, Ashland, OR, USA) was used to perform the analysis. At least 100,000 events were acquired for each samples.

### Statistical analyses

We examined the percentages and expression of biomarkers on distinct subsets of T cells, MAIT and Tfh cells between the four study groups. For multiple group comparisons, categorical variables were examined using the Chi-square test of Fisher’s Exact Test, while continuous variables were tested using non–parametric Kruskal–Wallis Test. If the P values were < 0.05, four–way comparisons were made between the four patient groups using the Mann–Whitney Test. The Spearman’s Rank correlation was used to compare the correlation between two continuous variables. The association between the surface markers, and functional markers and plasma PVL were assessed using the linear regression model. *P < 0.05, **<0.01, ***<0.001, and ****<0.0001 were used to determine significance. GraphPad Prism Ver.6.0 software (GraphPad, La Jolla, CA, USA) was used to perform all the analyses.

## Results

### Clinico-demographic characteristics of participants

The four groups, non-randomized study design consisted of 38 individuals. Thirteen subjects with chronic HBV infection who tested positive for HBsAg, anti-HBc as well as HBV DNA: Group 1 (G1), eight subjects with HCV RNA positive and anti-HCV positive; G2, seven subjects with HIV RNA positive, and 10 healthy controls (HC) (G4). The samples were collected between September and October of 2021. As per the analytical parameters, 54% (n=7) HBV-infected individuals, 87% (n=7) HCV-infected individuals, and 47.5% (n=3) HIV-infected individuals were diagnosed with signs of liver injury, while 46% (n=6) of the HBV-infected, 13% (n=1) of the HCV-infected, and 52.5% (n=4) of the HIV-infected participants were chronically infected without any underlying clinical or biochemical signs of liver injury (Table 1).

### Activated T cells with high PD-1 expression was observed in chronic HBV, HCV and HIV-infected subjects

**Figure 1:**
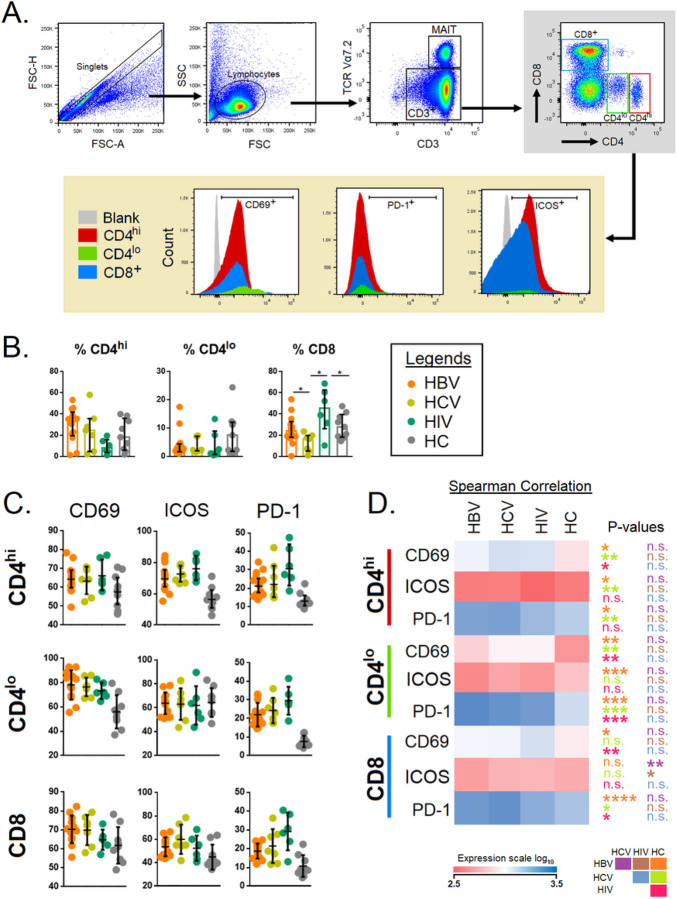
**A)** Gating strategy for CD69, ICOS and PD-1 expression on peripheral CD4^+hi^, CD4^lo^, and CD8^+^ T cell populations. Lymphocytes were gated from whole human PBMCs using height and area of forward scatter, then singlet gates were utilized to remove doublet populations. This was followed by lymphocyte gating using forward and side scatters areas. This was followed by a total CD3^+^ cell gate against TCR iVα7.2. From CD3^+^ cells, total CD8^+^ cells CD4^+hi^, and CD4^+lo^ were gated out. From this CD8^+^, CD4^+hi^, and CD4^+lo^ we determined CD69^+^, PD-1 and ICOS. **B)** The results of these gates are three T cell populations: CD8^+^, CD4^+hi^, and, CD4^+lo^. Comparison of the levels of, CD4^+lo^, CD4^+hi^ and CD8^+^ among patients chronically infected with HBV, HCV, HIV, and HCs. **C)** CD69, PD-1 and ICOS expression was determined by using a CD69, PD-1, and ICOS mean fluorescence intensity (MFI), respectively, which was used as a negative control for CD69, PD-1, and ICOS staining and allowed for accurate gating on the positive populations only. **D)** Expression (MFI) of CD69, ICOS, and PD-1 in CD4^+hi^, CD4^lo^ and CD8^+^ T cells among patients chronically-infected with HBV, HCV, HIV and HCs. The level of expression of each marker was reflected by the color scale of the heatmap. The cells were compared across the four groups by the Kruskal–Wallis test. *Post-hoc* Mann–Whitney U tests were subsequently performed only for those biomarkers with a Kruskal–Wallis test *P* value of <0.05. *P*<0.05 are considered significant; *P<0.05, **P<0.01, ***P<0.001, ****P<0.0001.

The flow cytometry gating strategy to delineate the CD4^+hi^, CD4^+lo^ and CD8^+^ T cells is depicted in Figure 1A. The CD8^+^ T cell levels were significantly higher for HIV (Figure 1B) as expected. There was negligible alteration in CD69, ICOS, or PD-1 (CD279)-expressing T cells between the different groups (Figure 1C-D). CD69 was significantly increased on CD4^+hi^ T cells in chronic HBV, HCV and HIV in comparison with HCs (p<0.05, p<0.01, and p<0.05, respectively). The expression of co-stimulatory (ICOS) and co-inhibitory (PD-1) markers were significantly higher in CD4^+hi^ T cells in HBV (p<0.05), HCV-infected (p<0.01) and HIV-infected (p<0.01) individuals. Likewise, CD69^+^CD4^+lo^ T cell levels (p<0.01) PD-1^+^CD4^+lo^ T cell levels and (p<0.001) were significantly higher in all infected groups whereas the levels of ICOS^+^CD4^+lo^ T cells were only significantly higher in chronic HBV (p<0.01) infection. CD69^+^CD8^+^ T cell levels were significantly increased in HBV (p<0.05) and HCV-infected individuals (p<0.01). Furthermore, all groups had significantly higher levels of PD-1^+^CD8^+^ T cells. (Figure 1C-D). Together, these data highlight that markers of T-cell activation coupled with exhaustion were increasingly expressed on T cells in chronic HBV, HCV and HIV infections.

### Circulating MAIT and Tfh cells represented activated phenotypes coupled with higher PD-1 expression in chronic HBV, HCV, and HIV-infected subjects

**Figure 2:**
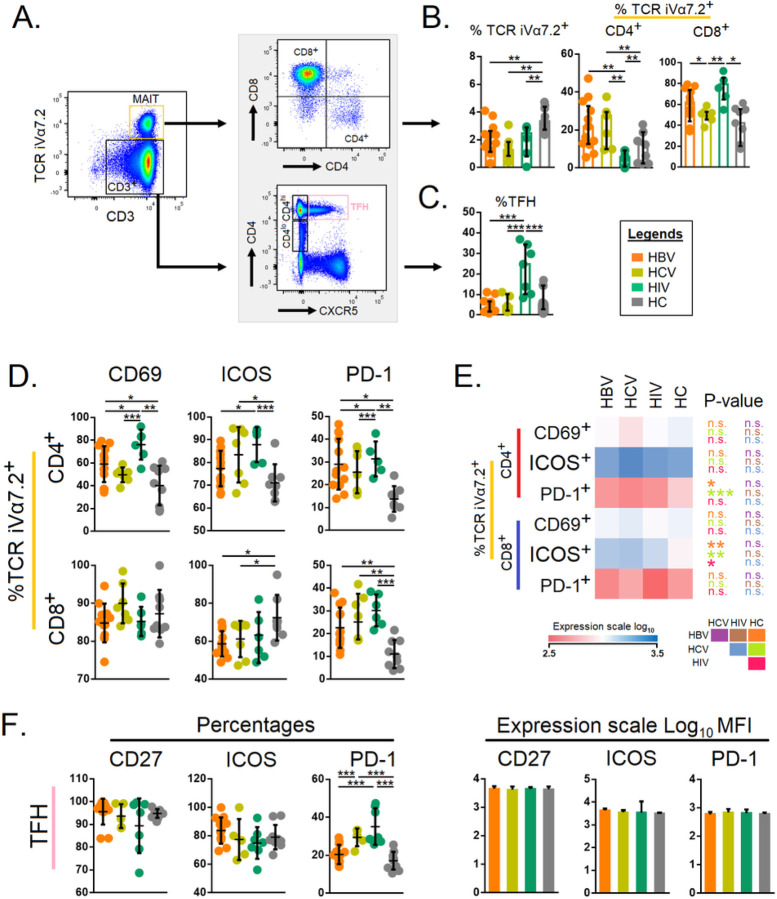
**A)** Gating strategies for mucosal-associated invariant T cells and follicular T helper cells. Total CD3^+^ cells were gated against TCR iVα7.2 (MAIT cells). From MAIT cells CD8^+^ and CD4^+^ T cell populations were gated. From CD3^+^ cells, CD4^+^ cells were gated against CXCR5. The different gates were determined: TCR iVα7.2, CD4^+^ TCR iVα7.2, CD8^+^ TCR iVα7.2, CD4^+^ CXCR5. **B)** Comparison of the levels of TCR iVα7.2, CD4^+^ TCR iVα7.2, CD8^+^ TCR iVα7.2 among individuals chronically infected with HBV, HCV, HIV, and HCs.**C)** Comparison of the levels of TFH among patients chronically infected with HBV, HCV, HIV, and HCs. **D)** Percentage level of CD4^+^ TCR iVα7.2, CD8^+^ TCR iVα7.2 were compared with CD69, ICOS expression and PD-1. **E)** Expression (mean fluorescence intensity, MFI) of CD69, ICOS, and PD-1 in CD4^+^ TCR iVα7.2, CD8^+^ TCR iVα7.2 with among individual chronically infected with HBV, HCV, HIV, and HCs. **F)** Percentage levels of TFH were compared with CD27, PD-1, and ICOS expression. G) The expression scale for MFI of CD27, ICOS and PD-1 in CD4^+^ TCR iVα7.2, CD8^+^ TCR iVα7.2 among individual chronically infected with HBV, HCV, HIV, and HCs. The level of expression of each marker was reflected by the color scale of the heatmap. The cells were compared across the four groups by the Kruskal–Wallis test. *Post-hoc* Mann–Whitney U tests were subsequently performed only for those biomarkers with a Kruskal–Wallis test *P* value <0.05. *P*<0.05 are considered significant; *P<0.05, **P<0.01, ***P<0.001, ****P<0.0001.

Next, we set out to study the MAIT and Tfh cells across the different study groups with a pre-determined gating strategy (Figure 2A). Total TCR iVα7.2^+^ MAIT cells were significantly lower in all the infected groups (p<0.01) compared with HCs. The CD4^+^MAIT cell levels were significantly increased in HCV and HBV groups (p<0.01) but not in the HIV-infected group compared to HCs. The level of CD8^+^MAIT cells (p<0.01) as compared to HCs were higher in all the groups (Figure 2B). The circulating Tfh cell levels in HIV-infected individuals were significantly higher (p<0.001) in HIV infected group, but comparable in the HBV and HCV-infected groups (Figure 2C). The CD69^+^CD4^+^MAIT cell levels were significantly increased in the HIV and HBV groups (<0.05) but not in the HCV-infected group. PD-1^+^CD4^+^MAIT cell levels were significantly elevated in all the infected groups (Figure 2D). The ICOS^+^CD8^+^ MAIT cells were significantly decreased for HCV and HBV groups, and the PD-1^+^CD8^+^ T cell levels were highly significant in HIV-infected individuals with p<0.001 (Figure 2D). Spearman correlation of CD4^+^ TCR iVα7.2, CD8^+^ TCR iVα7.2 with CD69, ICOS, and PD-1 is presented in Figure 2E. The functional markers for Tfh viz., CD27, ICOS and PD-1 were comparable between HBV, HCV and HCs. Nonetheless, PD-1 was highly elevated in the HIV-infected group suggesting that during chronic HIV infection the Tfh cells become activated (see Figure F-G).

### Significant elevation in cytokine-producing T cells during chronic viral infection

**Figure 3:**
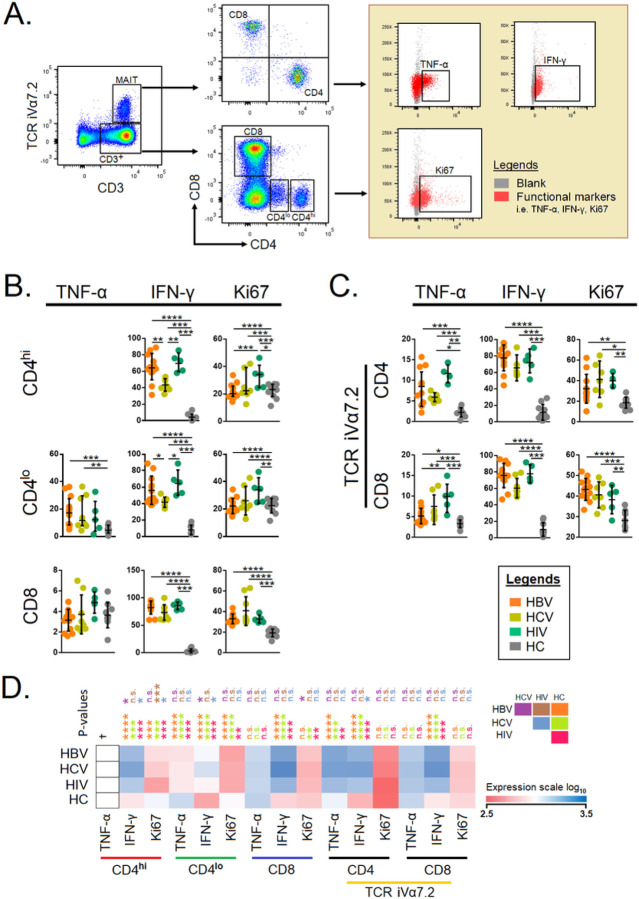
**A)** Gating strategies of intercellular cytokines in conventional and unconventional T cells. Total CD3^+^ cells were gated against TCR iVα7.2 (MAIT cells). From MAIT cells CD8^+^ and CD4^+^ T cell populations were gated whereas CD3^+^ was gated as CD4^+hi^, CD4^+lo^, and CD8^+^ T cells. The functional markers of different gates were determined: CD4^+^ TCR iVα7.2, CD8^+^ TCR iVα7.2, CD4^+^, and CD8^+^ cells **B)** Comparison of the levels of TNF-α, IFN-γ, and Ki67 in CD4^+hi^, CD4^+lo^ and CD8^+^ T cells among individuals chronically-infected with HBV, HCV, HIV, and HCs. **C)** Comparison of the levels of CD4^+^ MAIT cells, and CD8^+^ MAIT cells among patients chronically-infected with HBV, HCV, HIV, and HCs. **D)** The level of expression of TNF-α, IFN-γ, Ki67 with CD4^+hi^, CD4^+lo^, CD8^+^, CD4^+^ TCR iVα7.2, CD8^+^ TCR iVα7.2. The level of expression for each marker was reflected by the color scale of the heatmap. The cells were compared across the four groups by the Kruskal–Wallis test. *Post-hoc* Mann–Whitney U tests were subsequently performed only for those with a Kruskal–Wallis test P value of <0.05. P<0.05 are considered significant; *P<0.05, **P<0.01, ***P<0.001, ****P<0.0001. †, TNF-α did not express in the CD4^hi^ population.

Next, we compared the cytokine-producing ability of T cells with respect to CD4^+hi^, CD4^+lo^, CD8^+^ T cells and compared them with HCs results indicates that there is a significant increase in the cytokine^+^ CD4^+^ T cells and CD8^+^ T cells during chronic viral infection when compared to HCs. The comparison analysis of CD4^+hi^ IFN-γ was significant in HBV and HIV-infected individuals (p<0.0001). Ki67^+^CD4^+hi^ was significantly higher in HIV-infected individuals (p<0.0001). CD4^+lo^TNF-α was significant in HCV-infected individuals (p<0.001). CD4^+lo^IFN-γ and Ki67was highly significant in HIV (p<0.0001) infection. CD8^+^ IFN-γ and Ki67 had significance (p<0.0001) in HBV and HCV-infected subjects, respectively (Figure 3A and 3B).

Next, we also looked at the proliferating potential of T cells in the same assay, which indicates that Ki67^+^ T cells were higher in chronic viral infections as compared to HCs. Then, we examined the production of cytokines by the CD4^+^ and CD8^+^ MAIT cells. Both CD4^+^ and CD8^+^ MAIT cells expressed higher levels of TNF-α, and IFN-γ, together with higher proliferating ability as compared to HCs. They displayed significance (p<0.05) and (p<0.0001) in HIV- and HBV-infected individuals, respectively. The proliferating MAIT cells, Ki67^+^CD4^+^MAIT and Ki67^+^CD8^+^ MAIT cells, showed more significance in HIV and HBV-infected individuals (Figure 3C) compared to the HCV-infected group. Spearman correlation analysis and the significance of various cytokines in chronic HBV, HCV and HIV-infected individuals are shown in Figure 3D.

### Similar expression profile of markers across individuals with chronic HBV, HCV and HIV infections

**Figure 4:**
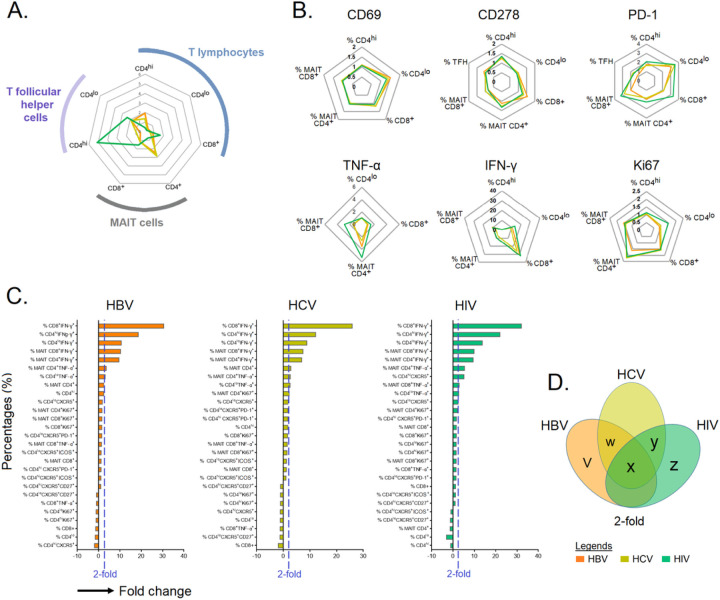
Immune cell profiling in chronic HBV-, HCV-, and HIV-infected individuals. **A-B)** The fold change in immune cells with identified intracellular and extracellular markers in individuals chronically infected with HBV, HCV and HIV normalized against HCs. **C)** Bar plot depicting mean 2-fold change among identified markers in specific immune cell type. **D)** Venn diagram showing immune cells that are upregulated >2-fold. The Venn diagram identified a profile of seven immune cell populations that are commonly expressed among chronically HBV-, HCV-, and HIV-infected individuals. **Footnotes: v.** %CD4^hi^; **w.** %MAIT CD4^+^; **x.** %CD4^lo^ TNF-α^+^, %MAIT CD4^+^ TNF-α^+^, %MAIT CD4^+^ IFN-γ^+^, %MAIT CD8^+^ IFN-γ^+^, %CD4^hi^ IFN-γ^+^, %CD4^lo^ IFN-γ^+^, CD8^+^ IFN-γ^+^; **y**. %MAIT CD4^+^ Ki67^+^, %CD4^lo^ CXCR5^+^; **z**. %CD4^hi^ CXCR5^+^, %MAIT CD8^+^ TNF-α^+^

In this study, we observed that the infected groups shared a similar activation and cytokine profiles with total CD4^+^, CD8^+^ T cells along with Tfh, and MAIT cells being the principal cells showing elevated activation and cytokine levels (Figure 4A and 4B). The cells that elevated >2-fold for each chronic infection were identified and displayed in Venn diagrams after the cytokine fold change was graded by descending order (Figures 4C and 4D). The analysis revealed that among all the immune cells, seven viz., CD4^+lo^TNF-α, MAIT CD4^+^ TNF-α, MAIT CD4^+^ IFN-γ, MAIT CD8^+^ IFN-γ,CD4^+lo^IFN-γ, CD4^+hi^ IFN-γ, CD8^+^ IFN-γ were common among patients chronically-infected with HBV, HCV, and HIV. Ki67^+^MAIT CD4^+^ and CXCR5 CD4^+lo^ was common among those chronically infected with HCV and HIV. CD4^+^MAIT was common among chronic HBV and HCV-infected individuals. The bar chart describes the %fold change of each cell subset (see Figure 4C-D and Supplementary Figure 1). The activation, exhaustion and functional markers in association with viral load of T-cell subsets are presented in Table 2.

### Increased CD69 expression on MAIT cells correlates with low plasma viral loads in HBV, HCV and HIV infections

**Figure 5:**
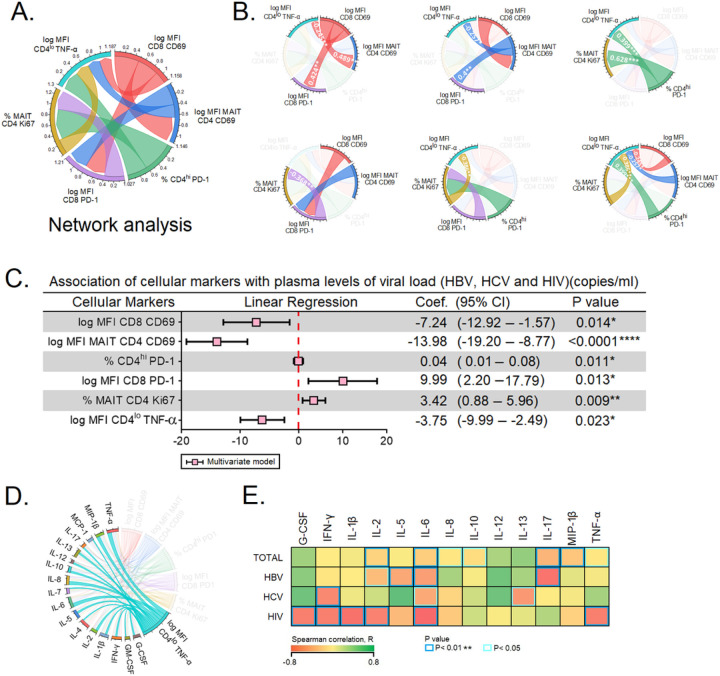
Predictors of plasma viral load. **A-B)** Network analysis of the six predictors of plasma viral load. Panel A depicts the complexity of the interactions between the six predictors. **B)** Spearman correlation between the six predictors of plasma viral load. **C)** The six markers were subjected to multivariate linear regression analysis to determine the markers that independently predict the plasma viral load. **D)** Expression of TNF-α and their association with plasma cytokines. **E)** Plasma level of cytokines associated with plasma viral load. Variables with P values <0.05 were considered independent predictors in their respective models. *, **, **** represent P<0.05, <0.01, and <0.0001, respectively.

We performed a network analysis for the six predictors of PVL, viz., CD4^+lo^TNF-α, CD69^+^CD8^+^, CD69^+^CD4^+^MAIT, PD-1^+^CD4^+hi^, PD-1^+^CD8^+^, Ki67^+^CD4^+^MAIT cells (Figure 5A). CD69^+^CD8^+^ showed a positive correlation with CD69^+^ CD4^+^MAIT, PD-1^+^CD8^+^, and a negative correlation with CD4^+lo^TNF-α. Further, CD69^+^CD4^+^MAIT had a positive correlation with PD-1^+^CD8^+^ and a negative correlation with CD4^+lo^TNF-α. Moreover, PD-1^+^CD4^+hi^ also showed a positive correlation with Ki67^+^CD4^+^ and CD4^+lo^TNF-α. We also found that PD-1^+^CD8^+^ were positively correlated with Ki67^+^CD4^+^. Ki67^+^CD4^+^MAIT were negatively correlated with CD4^+lo^TNF-α. In short, CD4^+lo^TNF-α had a positive correlation with PD-1^+^CD4^+hi^ and a negative correlation with CD69^+^CD8^+^, CD69^+^ CD4^+^MAIT, and Ki67^+^CD4^+^MAIT (Figure 5B). Together, we found that the markers associated with immune activation, proliferation and exhaustion were differentially expressed and correlated with the different T cell phenotypes.

### Inverse association of cellular markers with plasma levels of viral load in chronic HBV, HCV and HIV

Next, we performed multivariate linear regression analysis among the six subsets of T cells to determine independent prediction over the PVLs of HBV, HCV and HIV-infected individuals. The multivariate linear regression analysis of cellular markers were as follows: CD69^+^CD8^+^, −7.24 (p=0.014), CD69^+^CD4^+^MAIT, −13.98 (p=0.0001), PD-1^+^CD4^+hi^, 0.04 (p=0.011), PD-1^+^CD8^+^, 9.99 (p=0.013), Ki67^+^CD4^+^MAIT, 3.42 (p=0.009) and CD4^+lo^TNF-α, −3.75 (p=0.023) (Figure 5C). We also determined the expression of CD4^+lo^TNF-α and its association with plasma cytokines. We observed that CD4^+lo^TNF-α was significant among almost all the cytokines (Figure 5D). The PVLs across all the three viral infections were associated with various cytokines of which 13 revealed positive correlations (Figure 5E). These 13 cytokines, in turn, had an inverse correlation with the PVL. In HBV PVL IL-5, IL-6, and IL-17 were significant (p<0.01), and IL-2 (p<0.05) with a negative correlation. In comparison with HCV PVL, IFN-γ was significant (p<0.01); IL-6 and IL-13 also revealed significance (p<0.05) with a negative correlation. In the case of HIV PVL, G-CSF, IL-1β, IL-2, IFN-γ, IL-6, and TNF-α were negatively significant (p<0.01). Among all the infected individuals IL-17, and MIP-1β were inversely significant (p<0.01): IL-8, IL −10, TNF-α (p<0.05). Together, our data suggest that the inverse correlation of cellular markers may independently play a vital role in determining the plasma viral load of chronic HBV, HCV and HIV-infected patients.

## Discussion

Virus-specific T cells express multiple inhibitory receptors during chronic infections eventually impairing T cell functions. Studies in murine [11] simian [12] and humans have shown that blockade of the inhibitory molecules restores immune functions *in vitro* as well as *in vivo* [9]. CD4^+^ T cells provide help to other effector cells, especially CD8^+^ T cells to aid in their activation and cytokine production (TNF-α and IFN-γ), and contact-dependent cytotoxicity via perforin/granzyme synthesis and/or Fas-FasL interactions to render in viral elimination [13]. Chronic viral infections frequently result in decreased CD8^+^ T cell functions as compared to the potent effector T cells activated during acute infections [14]. Exhausted T cells in chronic infections can be classified into terminally-exhausted CD8^+^ T cells, and cells with a preserved ability to proliferate [15]. Exhausted T cells along with TNF-α, and IFN-γ induce non-specific immune responses aiding in retaining the infection [9].

Recent evidence suggest that MAIT cells are key to immune surveillance, especially in chronic viral infections. MAIT cells aid in host defense in an antigen-independent manner, as they respond to a variety of cytokines such as IL-12 and IL-18 [16] during viral infections. In chronic HBV and HIV infections plasma IL-5 and IL-7, respectively may play a significant role in viral suppression [17]. MAIT cells have been shown to act against several viral agents, particularly HBV, HCV, HIV, dengue, and influenza viruses [18,19]. Although available findings portray their significance in chronic HBV, HCV and HIV infections, very few compared their role in cross-sectional studies conducted across the infections from same region. In addition, we have also explored into the activation and functional status of circulating Tfh cells across the different study groups.

In the current study, we observed the activation and proliferation of MAIT cells with higher expression of CD69 and Ki67, respectively. Similar findings have also been observed during chronic HCV infection, where they exhibit an activated phenotype with higher levels of the activation markers CD69, HLA-DR, and CD38 [20]. Importantly, here we found that the co-stimulatory potential of MAIT cells were elevated in the three chronic viral infections, as these cells expressed higher levels of ICOS as compared to HCs. The correlation between CD69 levels and polyfunctional TCR iVα7.2^+^CD4^+^ T cells points to the critical role of CD69 against chronic HBV infection [21]. Similarly, here we observed that the levels of CD69-expressing CD8^+^ T cells and MAIT CD4^+^ cells were associated with a decrease in PVL indicating the likely importance of CD69 in viral control. The major limitation of our study is that HBV and HCV are hepatotropic viruses, whereas HIV is lympho-tropic macrophage-tropic, and these distinct virus tropisms should have had an impact on the phenotypes of various T cell subsets during the chronic phase, which have not been contemplated in the current work.

Experiments conducted thus far on CD4^+^ T cells during chronic infections have focused on Th1, and only recently investigators have started looking at Tfh cells, in particular the circulating Tfh cells. Tfh, a diverse group of CD4^+^ T cell subsets are essential for complete B cell responses that include germinal center (GC) reactions, isotype-switching, and affinity maturation [22]. Data available from all the chronic infections that we have studied herein have shown evidence on the induction of Tfh cells. However, the circulating Tfh activation, proliferation and their cytokine profiles have seldom been studied in head-to-head comparisons. Similar to the MAIT cells reported herein, we also observed increased activation of Tfh cells along with higher proliferation (Ki67), and co-stimulation with higher levels of ICOS expression. In all the chronic infections studied herein, we have shown evidence for higher levels of Tfh cells compared to HCs.

## Conclusions

In summary, our findings suggest a possible role of phenotypic markers as biological indicators of disease progression in chronic HBV, HCV, and HIV infections with their close association with disease severity, immune responses, and viral persistence. The upregulation of CD69 may aid in regulating immune response by determining the patterns of cytokine and chemokine release as well as the activation of lymphocytes during chronic infections. Enhanced TNF-α levels during chronic infection likely imply their protective role in viral elimination possibly via recruiting T cells.

## Supplementary Material

Supplement 1

## Figures and Tables

**Table 1:** Clinico-demographic characteristics of study participants

Characteristics	Total (n = 38)	HBV (n = 13)	HCV (n = 8)	HIV (n = 7)	HC (n = 10)	P value
Age, years	43 (32.5–58.0)	55 (33.3–67)	53.5 (45–65.5)	34 (32–42)	34 (28–42)	0.103
Sex, male (%)	24 (63.2%)	10 (76.9%)	6 (75%)	3 (42.9%)	5 (50%)	0.274
SGOT, U/L^†^	37 (27.1–66)	33.3 (21.4–75.2)	59.8 (40.7–68.2)	36.7 (30.7–48.2)	…	0.126
SGPT, U/L^†^	15.2 (10.5–26.2)	12.3 (5.4–13.4)	23.7 (17.8–47.8)	16.9 (12–21.6)	…	0.010*
GGT, U/L^†^	41.5 (18.4–93.9)	18.6 (13.4–71.2)	68.8 (37.6–117)	55.5 (33.1–93.4)	…	0.113
ALP, U/L^†^	58.4 (45.3–66.5)	52.7 (32.5–66.1)	63.7 (55.7–86.7)	59.5 (47.9–64)	…	0.188
PVL, copy/µL^†^	4.03 (1.63–4.64)	2.1 (1.46–2.5)	4.67 (1.9–4.78)	4.63 (4.6–6.66)	…	0.086
CD4^+^ T cell count, cells/µL	…	…	…	333 (241–265)	…	…

All data reported as median and interquartile range (IQR) unless specified. n, numbers; %, percentages; IQR, interquartile range; HBV, hepatitis B virus; HCV, hepatitis C virus; HIV, human immunodeficiency virus; HC, healthy control; PVL, plasma viral load; SGOT, serum glutamic oxaloacetic transaminase; SGPT, serum glutamic-pyruvic transaminase; GGT, gamma-glutamyl transferase; ALP, alkaline phosphatase.

* represents p < 0.05

† The median (IQR) was calculated in HBV, HCV, and HIV-infected individuals only.

**Table 2:** Activation, exhaustion and functional markers associated with plasma viral load

Marker	Univariate		Multivariate	
	Coef. (95% CI)	P value	Coef. (95% CI)	P value
**Demographic**				
**Age,** years	−0.002 (−0.022 – 0.018)	0.849	--	--
**Gender**, male	0.244 (−0.729 – 1.216)	0.617	--	--
**Activation markers**				
% CD4^hi^ CD69	0.006 (−0.060 – 0.071)	0.863	--	--
% CD4^hi^ ICOS	−0.021 (−0.075 – 0.034)	0.450	--	--
% CD4^lo^ CD69	−0.054 (−0.102 – 0.007)	0.025*	--	--
% CD4^lo^ ICOS	−0.043 (−0.075 – −0.010)	0.012*	--	--
% CD8 CD69	−0.043 (−0.110 – 0.024)	0.199	--	--
% CD8 ICOS	−0.036 (−0.077 – −0.005)	0.041	--	--
% MAIT CD4 CD69	−0.011 (−0.070 – 0.048)	0.706	--	--
% MAIT CD4 ICOS	0.029 (−0.022 – 0.079)	0.262	--	--
% MAIT CD8 CD69	−0.010 (−0.070 – 0.091)	0.799	--	--
% MAIT CD8 ICOS	−0.023 (−0.050 – 0.003)	0.040*	--	--
% TFH CD27	−0.042 (−0.104 – 0.019)	0.173	--	--
% TFH ICOS	−0.015 (−0.060 – 0.030)	0.499	--	--
log MFI CD4^hi^ CD69	−1.942 (−8.766 – 4.882)	0.570	--	--
log MFI CD4^hi^ ICOS	5.457 (0.463 – 11.377)	0.040*	--	--
log MFI CD4^lo^ CD69	−5.135 (−9.189 – −1.081)	0.014*	--	--
log MFI CD4^lo^ ICOS	−3.891 (−9.766 – 1.984)	0.189	--	--
log MFI CD8 CD69	−5.759 (−11.744 – −0.226)	0.039*	−10.066 (−15.148 – −4.984)	<0.0001****
log MFI CD8 ICOS	−1.759 (−11.4 – 7.883)	0.716	--	--
log MFI MAIT CD4 CD69	5.602 (1.359 – 9.844)	0.011*	−8.725 (−12.431 – −5.019)	<0.0001****
log MFI MAIT CD4 ICOS	0.709 (−1.992 – 3.411)	0.600	--	--
log MFI MAIT CD8 CD69	−3.214 (−7.058 – −0.631)	0.037*	--	--
log MFI MAIT CD8 ICOS	−2.251 (−4.868 – −0.365)	0.040*	--	--
log MFI MAIT TFH CD27	0.255 (−5.456 – 5.967)	0.929	--	--
log MFI MAIT TFH ICOS	−1.564 (−4.093 – 0.966)	0.220	--	--
**Exhaustion markers**				
% CD4^hi^ PD-1	0.059 (0.015 – 0.103)	0.009**	0.055 (0.034 – 0.077)	<0.0001****
% CD4^lo^ PD-1	0.003 (−0.055 – 0.060)	0.930	--	--
% CD8 PD-1	0.036 (−0.023 – 0.096)	0.224	--	--
% MAIT CD4 PD-1	0.037 (0.003 – 0.077)	0.041*	--	--
% MAIT CD8 PD-1	0.025 (−0.015 – 0.065)	0.209	--	--
% TFH PD-1	0.053 (0.006 – 0.100)	0.027	--	--
log MFI CD4^hi^ PD-1	2.363 (−2.885 – 7.611)	0.370	--	--
log MFI CD4^lo^ PD-1	5.860 (0.538 – 12.258)	0.042*	--	--
log MFI CD8 PD-1	−3.964 (−11.878 – 3.949)	0.319	--	--
log MFI CD4 PD-1	0.014 (−5.058 – 5.087)	0.996	--	--
log MFI CD8 PD-1	−2.618 (−4.480 – −0.755)	0.007**	5.468 (1.552 – 9.384)	0.007**
log MFI TFH PD-1	5.223 (0.303 – 10.142)	0.038*	--	--
**Functional markers**				
% CD4^hi^ TNF-α	0.103 (−0.124 – 0.330)	0.365	--	--
% CD4^hi^ IFN-γ	0.001 (−0.024 – 0.026)	0.949	--	--
% CD4^hi^ Ki67	0.059 (0.026 – 0.092)	0.001***	†	--
% CD4^lo^ TNF-α	0.017 (−0.024 – 0.057)	0.414	--	--
% CD4^lo^ IFN-γ	0.016 (−0.023 – 0.055)	0.410	--	--
% CD4^lo^ Ki67	0.013 (−0.022 – 0.048)	0.447	--	--
% CD8 TNF-α	−0.021 (−0.053 – 0.011)	0.191	--	--
% CD8 IFN-γ	−0.041 (−0.081 – −0.002)	0.042*	--	--
% CD8 Ki67 0.031	(−0.013 – 0.076)	0.164	--	--
% MAIT CD4 TNF-α	0.009 (−0.080 – 0.098)	0.840	--	--
% MAIT CD4 IFN-γ	0.008 (−0.015 – 0.031)	0.467	--	--
% MAIT CD4 Ki67	0.053 (0.026 – 0.079)	<0.0001****	0.034 (0.015 – 0.053)	<0.001***
% MAIT CD8 TNF-α	0.003 (−0.025 – 0.032)	0.806	--	--
% MAIT CD8 Ki67	0.001 (−0.055 – 0.056)	0.985	--	--
% MAIT CD8 IFN-γ 0.046	(−0.009 – 0.101)	0.101	--	--
log MFI CD4^hi^ TNF-α	−0.019 (−0.857 – 0.820)	0.964	--	--
log MFI CD4^hi^ IFN-γ	1.188 (−5.475 – 7.851)	0.720	--	--
log MFI CD4^hi^ Ki67	4.314 (1.968 – 6.659)	0.001***	†	--
log MFI CD4^lo^ TNF-α	−5.354 (−11.066 – −0.357)	0.045*	−7.794 (−13.400 – −2.188)	0.007**
log MFI CD4^lo^ IFN-γ	−0.148 (−3.729 – 3.434)	0.934	--	--
log MFI CD4^lo^ Ki67	−1.815 (−7.845 – 4.215)	0.546	--	--
log MFI CD8 TNF-α	−1.367 (−3.472 – 0.738)	0.197	--	--
log MFI CD8 IFN-γ	1.747 (−3.022 – 6.515)	0.463	--	--
log MFI CD8 Ki67	2.541 (−0.552 – 5.633)	0.104	--	--
log MFI MAIT CD4 TNF-α	4.39 (−3.233 – 12.030)	0.249	--	--
log MFI MAIT CD4 IFN-γ	1.817 (−2.528 – 6.162)	0.402	--	--
log MFI MAIT CD4 Ki67	4.632 (1.989 – 7.275)	0.001***	†	--
log MFI MAIT CD8 TNF-α	1.935 (−1.795 – 5.666)	0.300	--	--
log MFI MAIT CD8 Ki67	3.763 (0.177 – 7.703)	0.041*	--	--
log MFI MAIT CD8 IFN-γ	−1.671 (−4.186 – 0.844)	0.187	--	--

**Predictors for plasma viral load.** Univariate analyses by linear regression were performed to identify potential predictors of PVL. Given the small sample size, the linear regression modeling for T-cells subsets, activation, exhaustion and functional markers were performed separately. Variables with P <0.05 were considered as candidates. The candidate predictors were then included in a multivariate model, and variables with P values were <0.05 were considered as independent predictors in their respective models.

*, **, ***, **** represent P<0.05, <0.01, <0.001 and <0.0001, respectively.

†, the Ki67 expression of these subpopulations were in co-linearity with % MAIT CD4 Ki67. For all the Ki67+ subpopulation, only % MAIT CD4 Ki67 was included in multivariate analysis. Coef, coefficient.
