# Supplementary material for: Chronic viral infection compromises the quality of circulating mucosal-invariant T cells and follicular T helper cells via expression of both activating and inhibitory receptors
Source: Res Sq. 2023 Apr 27:rs.3.rs-2862719. Preprint. [Version 1] doi: 10.21203/rs.3.rs-2862719/v1 (PMC10168456; doi:10.21203/rs.3.rs-2862719/v1)
Supplement: Supplement 1 [file NIHPPRS2862719V1-supplement-1.pdf]

Supplementary Files

This is a list of supplementary files associated with this preprint. Click to download.

- SuppFig1.tif
